# Effect of Cr^3+^ doping on structural and optical properties of Eu^3+^ doped LaVO_4_ phosphor

**DOI:** 10.1039/d2ra06962h

**Published:** 2023-01-31

**Authors:** Ekta Rai, Ram Sagar Yadav, Dinesh Kumar, Akhilesh Kumar Singh, Vijay Janardhan Fulari, Shyam Bahadur Rai

**Affiliations:** a Department of Physics, Shivaji University Kolhapur 416004 India; b Department of Zoology, Institute of Science, Banaras Hindu University Varanasi 221005 India ramsagaryadav@gmail.com; c School of Materials Science and Technology, Indian Institute of Technology(Banaras Hindu University) Varanasi 221005 India; d Department of Physics, Institute of Science, Banaras Hindu University Varanasi 221005 India sbrai49@yahoo.co.in; e Department of Physics, National Post Graduate College Barhalganj Gorakhpur 273402 India

## Abstract

In this work, the Eu^3+^, Cr^3+^ doped and co-doped LaVO_4_ phosphors have been prepared through a high temperature solid-state reaction method. The powder XRD patterns of phosphors are very sharp and intense, which reflects a highly crystalline nature of phosphors. The XRD data were also refined by a Rietveld refinement method. The particle size of the phosphor samples lies in the sub-micron to micron range. The existence of La, Eu, Cr, V and O elements was verified by EDS spectra. The FTIR spectra show various absorption bands due to different vibrating groups. The optical band gap of the phosphor decreases on increasing concentration of Cr^3+^ ion. The photoluminescence excitation spectra of Eu^3+^, Cr^3+^ co-doped LaVO_4_ phosphor exhibit bands due to Eu^3+^ and Cr^3+^ ions. The Eu^3+^ doped LaVO_4_ phosphor exciting at 393 and 316 nm wavelengths gives intense red color at 614 nm due to the ^5^D_0_ → ^7^F_2_ transition of the Eu^3+^ ion. When the Cr^3+^ ion is co-doped in the Eu^3+^ doped LaVO_4_ phosphor the emission spectra contain emission bands due to Eu^3+^ and Cr^3+^ ions. The emission intensity of Eu^3+^ doped phosphor reduces due to energy transfer from Eu^3+^ to Cr^3+^ ions in presence of Cr^3+^ ions upon 393 and 386 nm excitations. The lifetime of the ^5^D_0_ level of Eu^3+^ ions decreases in the Eu^3+^, Cr^3+^ co-doped LaVO_4_ phosphor, which also reflects the energy transfer. The Eu^3+^, Cr^3+^ co-doped LaVO_4_ phosphor also produces a large amount of heat upon 980 nm excitation. Thus, the Eu^3+^, Cr^3+^ co-doped LaVO_4_ phosphors may be used for LEDs, solid state lighting and heat generating devices.

## Abstract 2

The Eu^3+^, Cr^3+^ co-doped LaVO_4_ phosphor gives red (614 nm) and NIR (700 nm) emissions due to Eu^3+^ and Cr^3+^ ions, respectively. The decrease in PL intensity of the Eu^3+^ doped LaVO_4_ phosphor is due to energy transfer from Eu^3+^ to Cr^3+^ ions.

## Graphical abstract

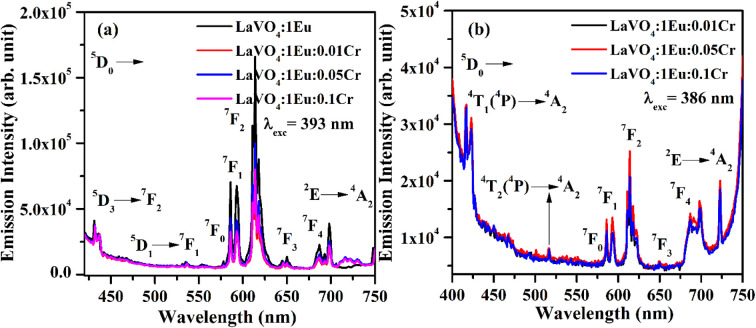

## 1. Introduction

The rare earth doped phosphor materials have been a subject of extensive investigations for several decades due to their wide applications in different fields in day-to-day life.^1–7^ They give intense sharp emissions in UV-vis-NIR regions and often lead to lasing, and Nd:YAG laser lasing at 1.06 μm is a good example of this.^8^ The intensity of this laser in NIR region is large enough so that the frequency doubled, tripled and quadrupled laser emissions could be obtained at 0.532, 0.355 and 0.266 μm, respectively and they are used for various applications. The rare earth ions attached with biomolecules are used as biosensors.^9,10^ The optical energy absorbed by biomolecules is transferred to the rare earth ion, which gives intense emission and thus, serves as a biosensor. The photoluminescence intensity of the rare earth ion in the host materials can be enhanced by co-doping of another neutral/ionized species acting as a sensitizer/modifier for the rare earth ions. In several cases, the sensitizer plays a dominant role.^11–15^ These materials have promising applications in different fields, such as display devices, light emitting diodes (LEDs), white LEDs, tunable color sources, optoelectronic devices, luminescent security ink, solar energy converters, optical heating, temperature sensing, bio-imaging, *etc*.^1–15^

The Eu^3+^ ion is a well-studied rare earth ion. It emits almost pure red color and often used in display devices, color televisions and computer screens. It also gives laser emission in the far-infrared region. The Eu^3+^ ion has been doped in large number of hosts including glass, micro- and nano-phosphors and also in the bio-mediums.^7,16–25^ The ground state of Eu^3+^ ion is ^7^F_*J*_ with ^7^F_0_ as the lowest one. The other component of it forms the low-lying excited states. The next low-lying excited state is regular ^5^D_*J*_ with ^5^D_0_ as the lowest one. The bands in visible region in it arise due to ^5^D_0, 1_ → ^7^F_*J*_ transitions. The Eu^3+^ ion emits very intense red band in almost all hosts in the range of 610–615 nm. The emitted radiation is mixed red because in many cases, it also emits intense orange at 590 nm and weak deep red near 650 nm and deep red at 700 nm. Ofcourse, the intensity of the red band is much larger than the orange band. Therefore, the Eu^3+^ doped materials are sometimes treated as pure red source of light for many practical purposes. It is also used to generate white light (RGB) with other rare earth/earths emitting green and blue colors.^1–6,16–25^ The ^5^D_0_ → ^7^F_1_ transition lies in orange region whereas ^5^D_0_ → ^7^F_3_ and ^5^D_0_ → ^7^F_4_ in deep red (NIR) regions. However, these bands are relatively weak. The band ^5^D_0_ → ^7^F_0_ appears with very small intensity and it is not seen in many cases. The ^5^D_0_ → ^7^F_2_ transition is due to change in electric dipole moment whereas the ^5^D_0_ → ^7^F_1_ transition occurs due to change in magnetic dipole moment. The ratio of the emission intensity of these bands *i.e.*, 
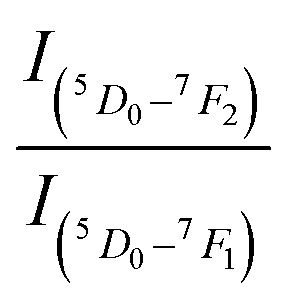
 gives the asymmetric nature of the host.^16–21^ The Eu^3+^ ion has been studied in different phosphor host matrices (ref. 26–31 and references therein) in which some of them are found to be self-activated.^26–29^

The LaVO_4_ as a self-activated host absorbs UV light efficiently. It emits blue color in the range of 350–450 nm with its maximum ∼430 nm. The Eu^3+^ ion absorbs this radiation and emits photoluminescence in the range of 420–750 nm. This induced us to select this as a host matrix.^32–34^ LaVO_4_ exists in monoclinic (m-LaVO_4_) as well as in tetragonal (t-LaVO_4_) phases. The formation of monoclinic or tetragonal phase depends on the temperature used for its preparation and also on the environment in which the samples have been prepared (in air or vacuum). Sometimes, it also depends on the doping of activator/sensitizer ions in the LaVO_4_ matrix.^25,27,28^ We have used 1473 K temperature for the preparation of phosphor samples. The XRD measurements clearly show that the phosphor samples exist in monoclinic phase at this temperature.^25,27,28,32–34^

The triply ionized Cr^3+^ ion with configuration 3d^3^ has been used as sensitizer as well as activators in different host matrices.^35–38^ The ^2^E state of Cr^3+^ ion is a long-lived state, which gives long lasting phosphorescence.^35,37^ It gives lasing at several wavelengths in IR and NIR regions in which the NIR emission at 695 nm is due to forbidden ^2^E → ^4^A_2_ transition and has been extensively studied in recent years.^38^ The Cr^3+^ ion also gives the blue (^4^T_1_(^4^P) → ^4^A_2_)/(^4^T_1_(^4^F) → ^4^A_2_), green (^4^T_2_(^4^P) → ^4^A_2_) and red (^4^T_2_(^4^F) → ^4^A_2_) emissions. It will be interesting to see that the energy level ^4^T_2_(^4^F) of Cr^3+^ ion lies in the red region at 15 773 cm^−1^ while that of ^4^T_1_(^4^F) lies in the green region at 17 211 cm^−1^. On the other hand, the ^5^D_0_ level of Eu^3+^ ion lies at 17 267 cm^−1^. Thus, when Eu^3+^ and Cr^3+^ ions are present in the host the ^4^T_1_(^4^F) of Cr^3+^ ion lies close but slightly below to the ^5^D_0_ level of Eu^3+^ ion. Thus, when Eu^3+^ ions are excited they transfer a small amount of their energy to Cr^3+^ ions and therefore, the emission intensity of Eu^3+^ ion as well as its lifetime are decreased. The presence of excess Cr^3+^ ion may also quench its own emission intensity.

In the present work, we have doped different concentrations of Cr^3+^ ions in the LaVO_4_:1 mol% Eu^3+^ phosphor. Initially, the Eu^3+^ doped LaVO_4_ has been studied and its concentration was optimized for optimum emission intensity. It has been found that the emission intensity is optimum at 1 mol% concentration of Eu^3+^ ion. Further, the samples were prepared using LaVO_4_:1 mol% Eu^3+^ phosphor with different concentrations of Cr^3+^ ions (where *x* = 0.01, 0.05, 0.1, 0.5, 1.0 mol%). We have studied the effect of Cr^3+^ doping on the absorption and photoluminescence emission intensity of LaVO_4_:1 mol% Eu^3+^ phosphor. It has been found that the emission intensity of LaVO_4_:1 mol% Eu^3+^ phosphor decreases even with small concentration of Cr^3+^ ions. We have also measured the lifetime of ^5^D_0_ level of Eu^3+^ ion corresponding to ^5^D_0_ → ^7^F_2_ band at 614 nm. The lifetime of ^5^D_0_ level initially increases and then decreased with the increase in the concentration of Eu^3+^ ion. Further, the lifetime of ^5^D_0_ level decreases on increasing the concentration of Cr^3+^ ion, which is due to energy transfer from Eu^3+^ to Cr^3+^ ions. This supports that the Cr^3+^ ion behaves as a quenching center. We have also measured the heat produced in the Eu^3+^, Cr^3+^ co-doped LaVO_4_ phosphor on excitation with 980 nm diode laser.

## 2. Experimental methods

### 2.1. Synthesis and characterization of samples

The La_(1−*x*)_Eu_*x*_VO_4_ (where *x* = 0, 0.5, 1.0, 1.5 and 2 mol%) and La_(1−*x*−*y*)_Eu_*x*_Cr_*y*_VO_4_ (where *x* is fixed at 1 mol% and *y* was varied as 0.01, 0.05 and 0.1 mol%) phosphor samples were synthesized by a high temperature solid-state reaction method at 1473 K. The raw materials *viz.* La_2_O_3_ (99.9% Himedia), V_2_O_5_ (99.9% Alpha Aesar), Eu_2_O_3_ (99.99% Merck) and Cr_2_O_3_ (99.99% alpha Aesar) were used as the starting materials. The calculated amounts of all the raw materials were weighed and mixed homogeneously with the help of an agate mortar and pestle for one hour each using acetone as mixing agent. The prepared mixtures were put in alumina crucibles and calcined in a high temperature furnace at 1473 K for 5 h. After that, the furnace temperature was reduced till it reaches to the room temperature and products were taken out from different crucibles. The phosphor samples were then crushed to convert them into fine powder form. The structural and optical measurements of these phosphor samples were carried out separately and compared with each other.

The XRD measurements of Eu^3+^, Cr^3+^ doped and co-doped LaVO_4_ phosphors monitored in the 2*θ* range of 10°–100° were carried out using MiniFlex 600 (Rigaku Japan) X-ray diffractometer at 2° per min scan speed with CuK_α_ as X-ray radiation source (*λ* = 1.5406 Å). The Rietveld refinements of all the XRD data were made using FullProf Suite.^32–34^ In order to measure the morphology and the energy dispersive X-ray spectroscopic (EDS) spectra of all the samples, a Zeiss EVO 18 Research unit of the scanning electron microscope (SEM) system was used. The absorption spectra of samples were recorded in the range in 200–800 nm using Lambda-750 PerkinElmer UV-vis-NIR spectrophotometer unit. The Fourier transform infrared (FTIR) spectra of samples were also measured with the help of Frontier-1 spectrometer, PerkinElmer unit. The photoluminescence excitation and emission spectra of the samples were monitored using Fluorolog-3 (Horiba) attached with 450 W Xenon lamp and photomultiplier tube (PMT). The lifetime of ^5^D_0_ level of the Eu^3+^ ion at 614 nm was also measured using the same unit in phosphorescence mode using 25 W pulsed Xenon lamp. The heat generated in all the samples were also measured using a thermo-couple set up at different power densities of 980 nm diode laser.

## 3. Results and discussion

### 3.1. Crystal structure and rietveld analysis

**Fig. 1:**
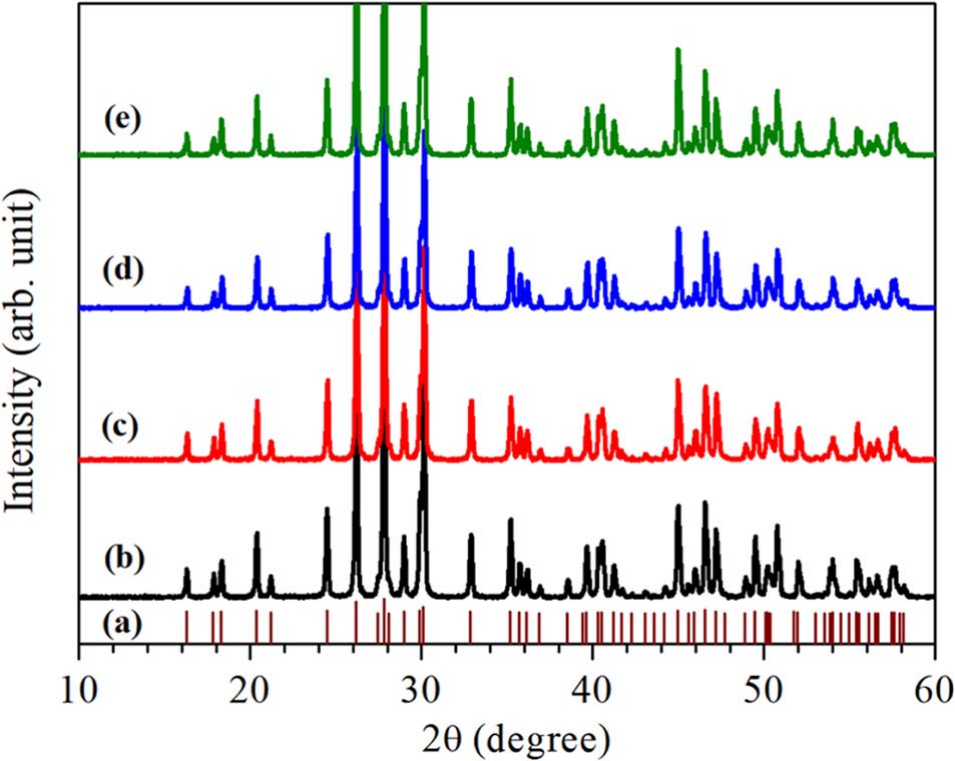
Room-temperature XRD patterns of Eu^3+^ and/or Cr^3+^ co-doped LaVO_4_ phosphors: (a) Standard XRD pattern of LaVO_4_ (JCPDF File No. 50-0367), (b) LaVO_4_:1Eu^3+^, (c) LaVO_4_:1Eu^3+^:0.01Cr^3+^, (d) LaVO_4_:1Eu^3+^:0.05Cr^3+^ and (e) LaVO_4_:1Eu^3+^:0.1Cr^3+^ phosphors.

For the structural analysis of LaVO_4_:1Eu^3+^:yCr^3+^ phosphors with *y* = 0.01, 0.05 and 0.1 mol%, the powder X-ray diffraction (XRD) patterns of these phosphor samples were monitored at room-temperature and are presented in Fig. 1. The powder XRD patterns of these phosphors are very sharp and highly intense, which reveal that the crystallinity of the LaVO_4_:1Eu^3+^:yCr^3+^ phosphors are very high.

The diffraction peaks in the powder XRD patterns of all the compounds, well match with the standard XRD data of monoclinic LaVO_4_ compound belonging to *P*2_1_/*n* space group with JCPDF File No. 50-0367 alongwith lattice parameters *a* = 7.0433(1) Å, *b* = 7.2806(2) Å, *c* = 6.7219(1) Å and *β* = 104.834(2)° (see Fig. 1). This implies that all Eu^3+^ and Cr^3+^ co-doped LaVO_4_ phosphors crystallize into monoclinic crystal structure belonging to *P*2_1_/*n* space group.^32–34^ No traces of any secondary phase in the XRD profile of these compounds are seen. The XRD patterns shift towards higher 2*θ* value with the increase of concentration of Cr^3+^ ion.

**Fig. 2:**
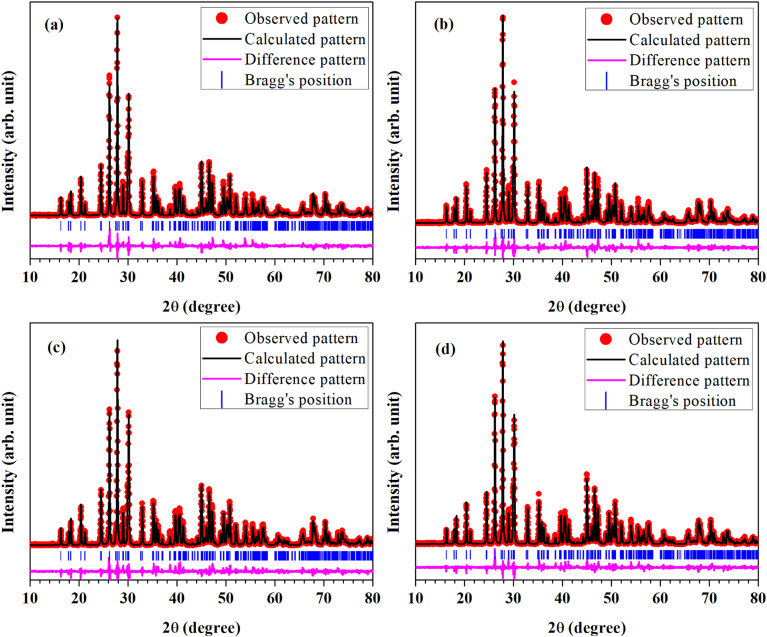
Rietveld fits of XRD patterns for Eu^3+^ and/or Cr^3+^ co-doped LaVO_4_ phosphors: (a) LaVO_4_:1Eu^3+^, (b) LaVO_4_:1Eu^3+^:0.01Cr^3+^, (c) LaVO_4_:1Eu^3+^:0.05Cr^3+^ and (d) LaVO_4_:1Eu^3+^:0.1Cr^3+^ phosphors.

In order to find out different crystal structure parameters, Rietveld structural analysis of the XRD patterns of all the phosphors have been carried out by the use of FullProf Suite.^39,40^ In the course of Rietveld structural analysis, we had supposed that the Eu^3+^ and/or Cr^3+^ ions are substituting at La-site in the LaVO_4_ host. In the monoclinic crystal structure having *P*2_1_/*n* space group, V^5+^, La^3+^/Eu^3+^/Cr^3+^, O^2−^(i), O^2−^(ii), O^2−^(iii) and O^2−^(iv) ions substitute at 4e site (δ*x*, δ*y*, δ*z*). Further, for Rietveld structural analysis, we have used atomic coordinates for constituent atoms as reported earlier.^32–34^ A good fit between the calculated and the observed XRD patterns was observed. Rietveld plots for the LaVO_4_:1Eu^3+^, LaVO_4_:1Eu^3+^:0.01Cr^3+^, LaVO_4_:1Eu^3+^:0.05Cr^3+^ and LaVO_4_:1Eu^3+^:0.1Cr^3+^ phosphors are displayed in Fig. 2a–d, respectively. In Fig. 2, solid red dots designate the observed XRD patterns and the continuous curve over the observed XRD patterns indicates the calculated patterns. The vertical bars in Rietveld fits demonstrate the positions of Bragg's peak and the lower most continuous curve signifies the difference among the observed and the calculated XRD patterns.

The obtained values of goodness of fit (*χ*^2^), lattice parameters (*a*, *b*, *c*, *α*, *β* and *γ*) and the unit cell volume (*V*) for all the doped and co-doped phosphor samples are given in Table 1. It has been observed that the value of unit cell volume decreases as the concentrations of Cr^3+^ ions are enhanced. Reduction in the unit cell volume of the Cr^3+^ doped LaVO_4_:1Eu^3+^ phosphors may be due to the smaller ionic radius of Cr^3+^ ion (0.615 Å) as compared to the ionic radius of La^3+^ ion (1.216 Å) and Eu^3+^ ion (1.120 Å).^34^ The Cr^3+^ ion would prefer to go at the La-site to maintain the charge balance in the LaVO_4_ phosphor.

**Table 1:** Refined value of goodness of fit (*χ*^2^), lattice parameters (*a*, *b*, *c*, *α*, *β* and *γ*) and the unit cell volume (*V*) of the monoclinic structure for Eu^3+^ and Eu^3+^ + Cr^3+^ doped and co-doped LaVO_4_ phosphors

Compound	Lattice parameters (*α* = *γ* = 90°)				Unit cell volume *V* (Å^3^)	*χ* ^2^
	*a* (Å)	*b* (Å)	*c* (Å)	*β* (°)		
LaVO_4_:1Eu^3+^	7.0388(2)	7.2771(2)	6.7200(2)	104.831(2)	332.75(2)	1.90
LaVO_4_:1Eu^3+^:0.01Cr^3+^	7.0393(1)	7.2766(1)	6.7194(1)	104.837(2)	332.71(1)	1.76
LaVO_4_:1Eu^3+^:0.05Cr^3+^	7.0389(2)	7.2762(2)	6.7195(2)	104.839(2)	332.67(2)	1.78
LaVO_4_:1Eu^3+^:0.1Cr^3+^	7.0390(2)	7.2760(2)	6.7191(2)	104.837(2)	332.65(2)	1.63

### 3.2. Microstructural analysis

**Fig. 3:**
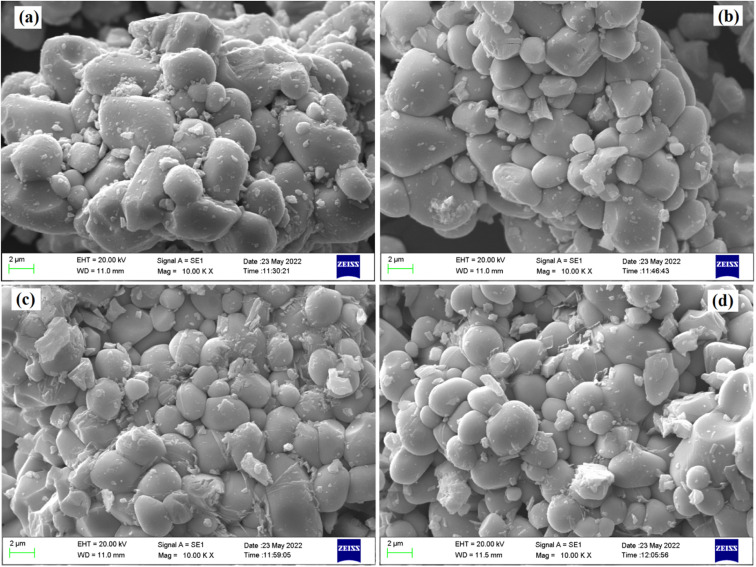
Scanning electron microscopic images for the Eu^3+^ and Eu^3+^ + Cr^3+^ co-doped LaVO_4_ phosphors; (a) LaVO_4_:1Eu^3+^, (b) LaVO_4_:1Eu^3+^:0.01Cr^3+^, (c) LaVO_4_:1Eu^3+^:0.05Cr^3+^ and (d) LaVO_4_:1Eu^3+^:0.1Cr^3+^ phosphors.

The microstructure of all the phosphor samples has been studied with the help of scanning electron microscopy (SEM) images, at a magnification of 10 K× and a scale of 2 μm. Fig. 3a–d displays SEM micrographs for the LaVO_4_:1Eu^3+^, LaVO_4_:1Eu^3+^:0.01Cr^3+^, LaVO_4_:1Eu^3+^:0.05Cr^3+^ and LaVO_4_:1Eu^3+^:0.1Cr^3+^ phosphor powder samples, respectively. The SEM images of the samples reveal that the particles size of the phosphor samples decreases as the doping concentration of the Cr^3+^ ions is increased. The particles of these phosphors are somewhat aggregated and most of the particles are spherical in shape (see Fig. 3). The particles size of the phosphor samples in general lies in the sub-micron to micron range; however, the average particles size in the present case is in the micron range. Thus, the average particles size of the phosphor samples has been calculated using ImageJ software. The mean value of the particles size was found to be 4.05, 3.47, 3.18 and 2.89 μm for the LaVO_4_:1Eu^3+^, LaVO_4_:1Eu^3+^:0.01Cr^3+^, LaVO_4_:1Eu^3+^:0.05Cr^3+^ and LaVO_4_:1Eu^3+^:0.1Cr^3+^ phosphors, respectively. This clearly shows that the particles size reduces on doping/co-doping of Eu^3+^ and Cr^3+^ ions.

### 3.3. Compositional analysis

**Fig. 4:**
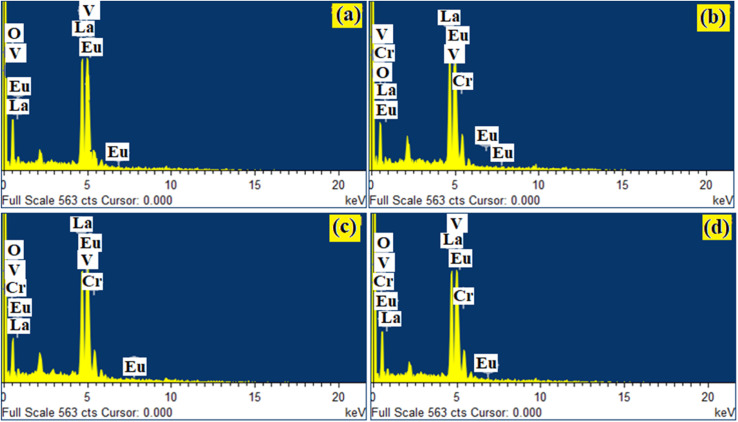
EDS spectra for Eu^3+^ and Eu^3+^ + Cr^3+^ co-doped LaVO_4_ phosphors *i.e.* (a) LaVO_4_:1Eu^3+^, (b) LaVO_4_:1Eu^3+^:0.01Cr^3+^, (c) LaVO_4_:1Eu^3+^:0.05Cr^3+^ and (d) LaVO_4_:1Eu^3+^:0.1Cr^3+^ phosphors.

Qualitative compositional analyses of the LaVO_4_:1Eu^3+^, LaVO_4_:1Eu^3+^:0.01Cr^3+^, LaVO_4_:1Eu^3+^:0.05Cr^3+^ and LaVO_4_:1Eu^3+^:0.1Cr^3+^ phosphor samples have been carried out by monitoring energy dispersive X-ray spectroscopy (EDS) spectra of the phosphor samples at the time during the SEM micrographs measurements. The EDS spectra of the LaVO_4_:1Eu^3+^, LaVO_4_:1Eu^3+^:0.01Cr^3+^, LaVO_4_:1Eu^3+^:0.05Cr^3+^ and LaVO_4_:1Eu^3+^:0.1Cr^3+^ phosphors are presented in Fig. 4a–d, respectively. The occurrence of the characteristic peaks corresponding to the component atoms, *i.e.*, La, Eu/Cr, V and O in the EDS spectra exhibits the presence of the component elements. As the EDS method is not suitable for the quantitative analysis of the light elements only qualitative analysis was performed.

**Fig. 5:**
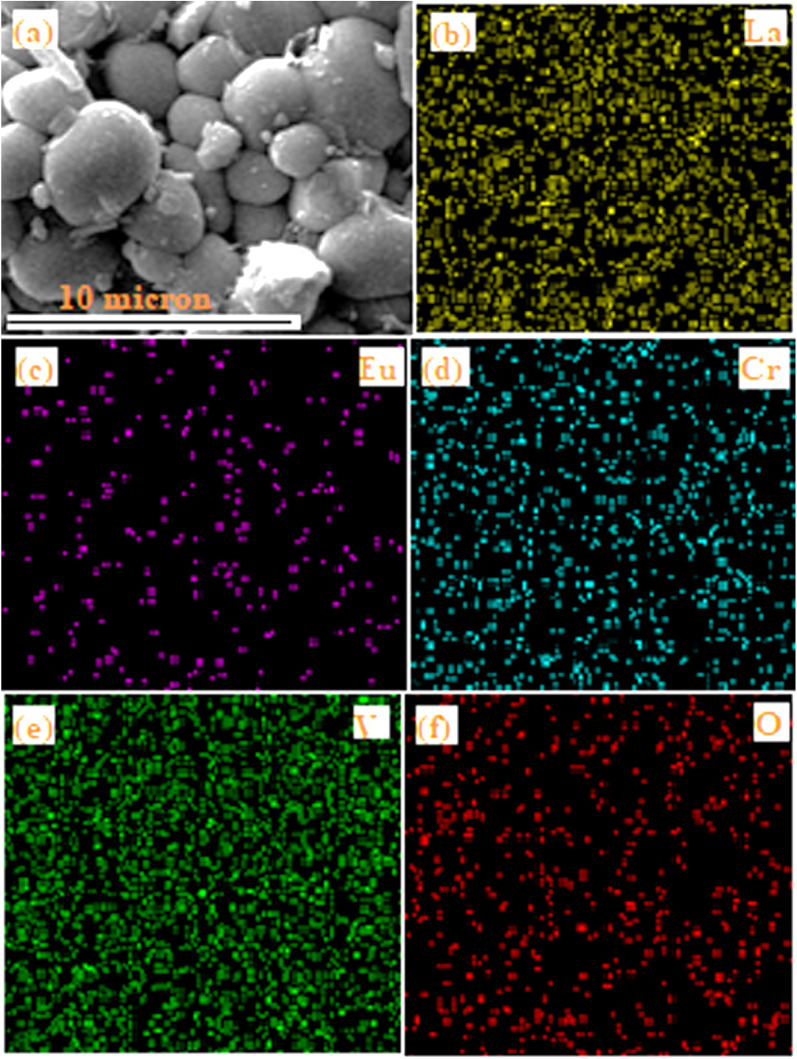
Elemental mapping and distribution of the component elements present in the LaVO_4_:1Eu^3+^:0.1Cr^3+^ phosphor: (a) selected area used for elemental mappings, (b) La, (c) Eu, (d) Cr, (e) V and (f) O elements.

Fig. 5 displays the selected area used for elemental mapping for the elements La, Eu, Cr, V and O present in the LaVO_4_:1Eu^3+^:0.1Cr^3+^ phosphor. The elemental mapping clearly demonstrates the homogeneous distribution of the elements (*i.e.*, La, Eu, Cr, V and O) present in the phosphor sample.

### 3.4. Optical measurements

#### 3.4.1 Fourier transform infrared (FTIR) spectra

**Fig. 6:**
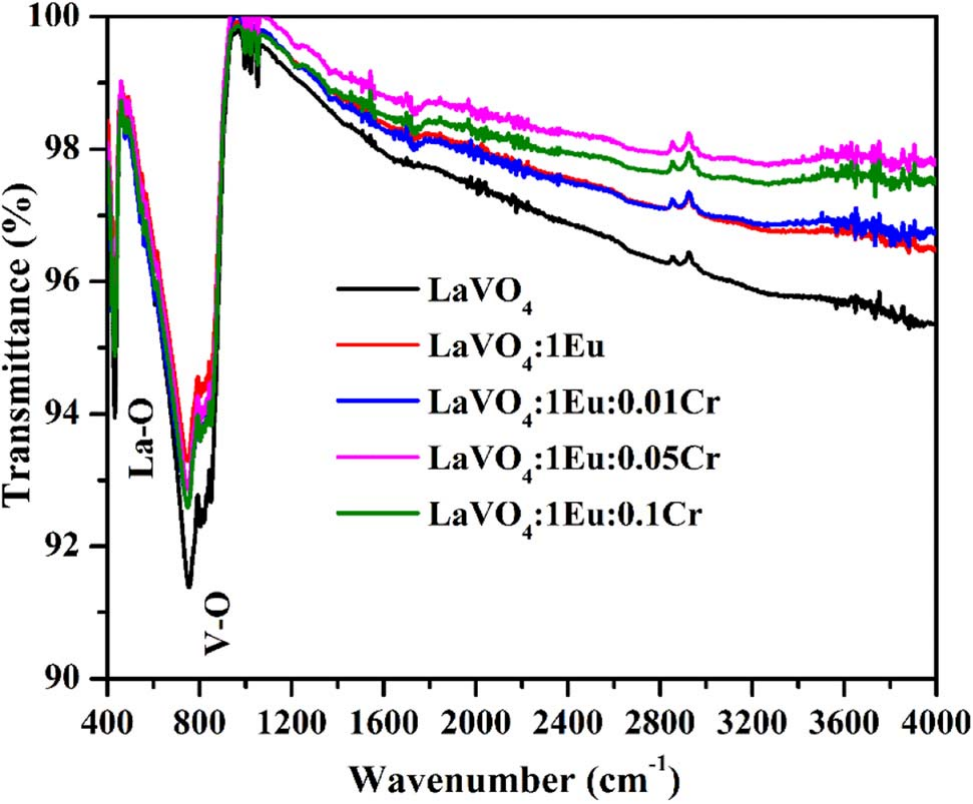
FTIR spectra of LaVO_4_, LaVO_4_:1Eu^3+^, LaVO_4_:1Eu^3+^:0.01Cr^3+^, LaVO_4_:1Eu^3+^:0.05Cr^3+^ and LaVO_4_:1Eu^3+^:0.1Cr^3+^ phosphor samples.

The FTIR spectra of the LaVO_4_, LaVO_4_:1Eu^3+^, LaVO_4_:1Eu^3+^:0.01Cr^3+^, LaVO_4_:1Eu^3+^:0.05Cr^3+^ and LaVO_4_:1Eu^3+^:0.1Cr^3+^ phosphor samples have been recorded in the spectral range of 400–4000 cm^−1^ and they are shown in Fig. 6. There is a variation in intensity of different absorption bands due to variation of charge distribution of Cr^3+^ and Eu^3+^ ions in the host. All the samples show three clear peaks near 431, 755 and 804 cm^−1^. These peaks are due to La–O stretching vibration; symmetric and asymmetric stretching vibrations of V–O bonds in VO_4_^3−^ group, respectively. In fact, there are large number of weak peaks near 804 cm^−1^ band due to VO_4_^3−^ group.^32–34^

#### 3.4.2 UV-vis absorption spectra of phosphor samples

**Fig. 7:**
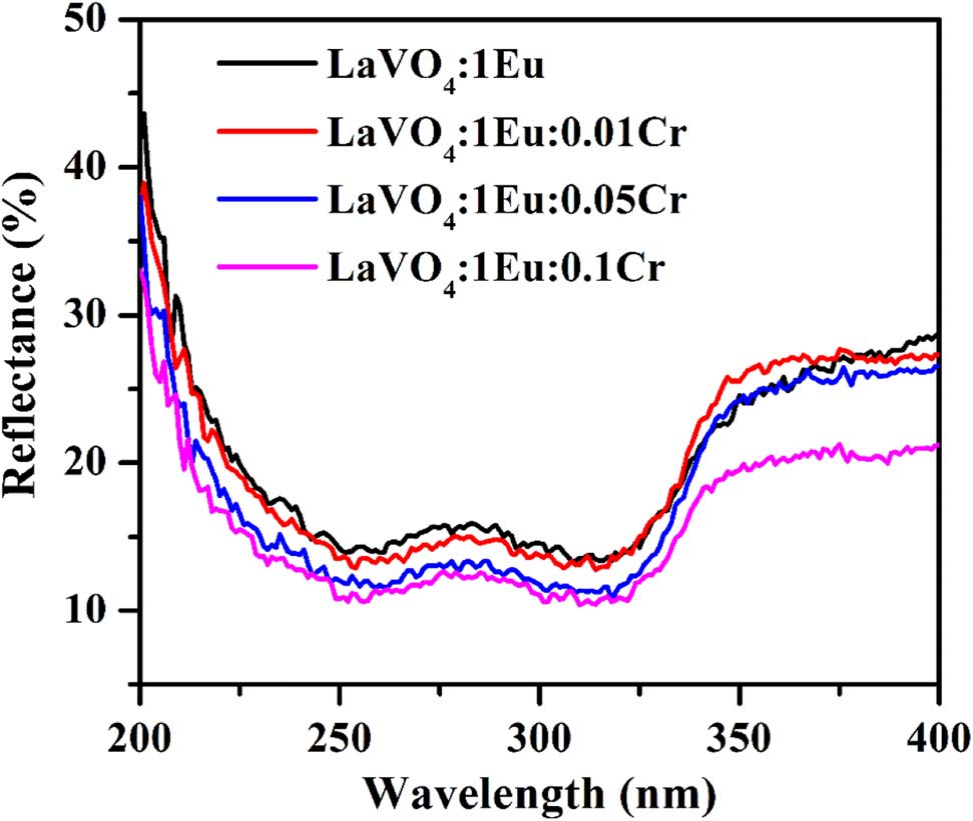
The UV-vis absorption spectra of LaVO_4_:1Eu^3+^, LaVO_4_:1Eu^3+^:0.01Cr^3+^, LaVO_4_:1Eu^3+^:0.05Cr^3+^ and LaVO_4_:1Eu^3+^:0.1Cr^3+^ phosphor samples.

The UV-vis absorption spectra of LaVO_4_:1Eu^3+^, LaVO_4_:1Eu^3+^:0.01Cr^3+^, LaVO_4_:1Eu^3+^:0.05Cr^3+^ and LaVO_4_:1Eu^3+^:0.1Cr^3+^ phosphor samples have been monitored in diffuse reflectance mode in the spectral region 200–400 nm and the resulting spectra are shown in Fig. 7. The spectra contain two intense broad bands at 254 and 316 nm. The intensity of these bands varies with Cr^3+^ concentrations; however, the peak positions remain unchanged. These bands arise due to charge transfer transition of O^2−^ → V^5+^ ions and from O^2−^ → Eu^3+^ ions in the LaVO_4_ phosphors. The doping of Cr^3+^ ion in the Eu^3+^ doped LaVO_4_ phosphor causes a field effect on the absorption intensity of O^2−^ → V^5+^ transition. The ground state of (VO_4_)^3−^ ion is ^1^A_2_(^1^T_1_). The observed bands arise due to transition from ground state to the close-lying electronic states ^1^A_1_(^1^E) and ^1^E(^1^T_1_). The charge transfer transition (CTS) of O^2−^ → Eu^3+^ also lies in 270–325 nm range and overlaps on (VO_4_)^3-^ bands.^17–19,32–34^

We have also monitored the absorption spectra of the samples in Kubelka–Munk (KM) mode to determine the optical band gap of LaVO_4_ and its dependence on different doping concentrations of Cr^3+^ ions. The Kubelka–Munk (KM) function is given as:^39–42^i
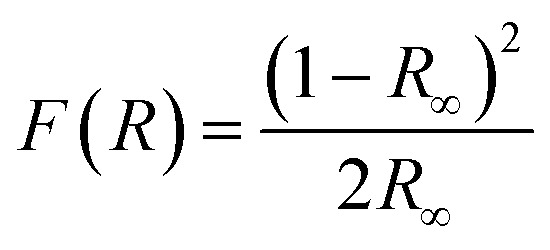
where, *R*_∞_ represents the diffuse reflectance intensity of the samples normalized to the non-absorbing standard samples. The KM function *F*(*R*) is related to the band gap (*E*_g_) as:ii
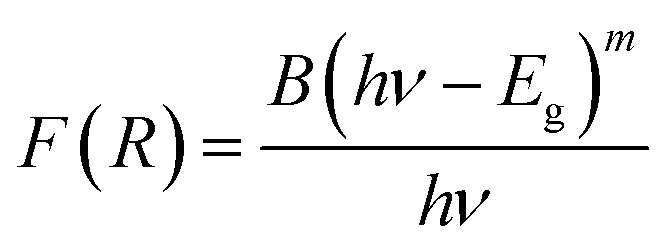


This relation is also called as Wood and Tauc formula (41) where *E*_g_, *hν* and *B* specify the optical band gap, energy of incident photon and the band tailoring parameter, respectively. The value of *m* is taken 
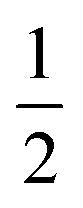
 supposing the case to be of direct optical band gap.^39–42^ Therefore,iii
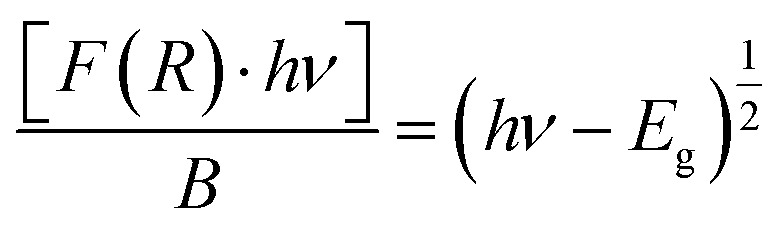


oriv
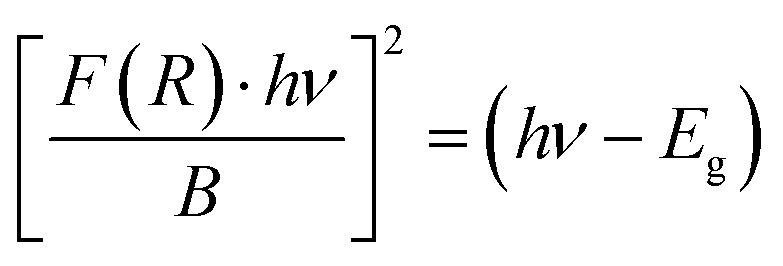
If we assume 
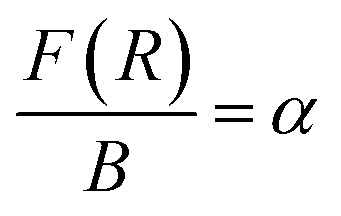
, then (*αhν*)^2^ = (*hν* − *E*_g_)

**Fig. 8:**
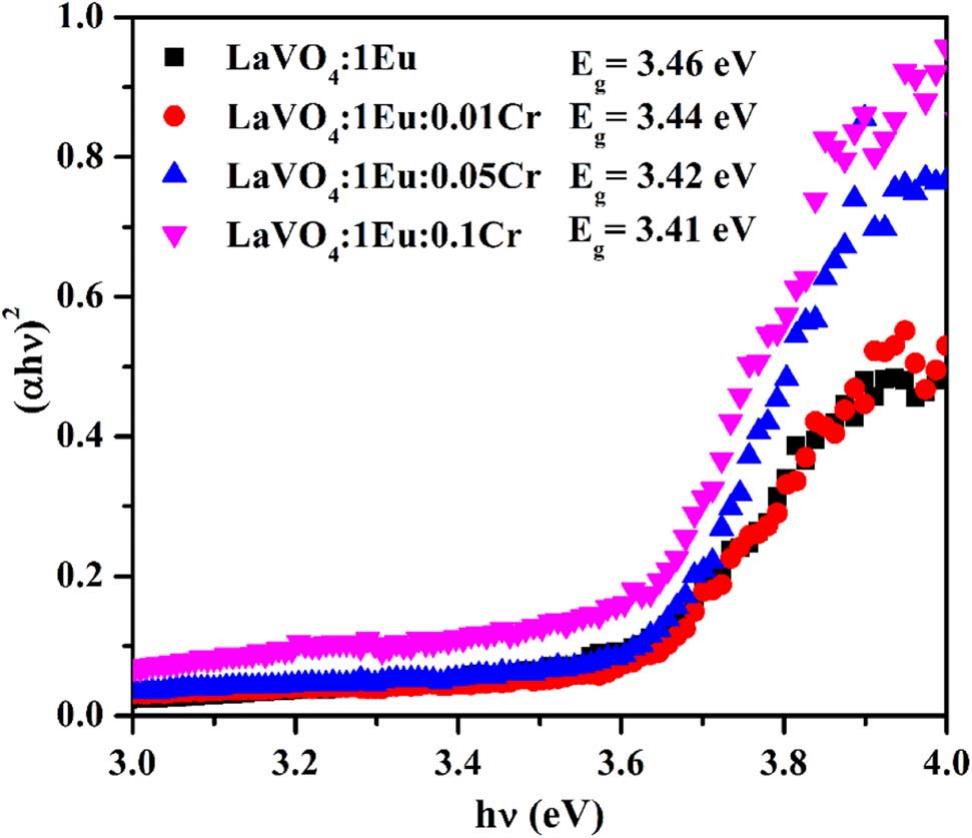
Optical band gap of LaVO_4_:1Eu^3+^, LaVO_4_:1Eu^3+^:0.01Cr^3+^, LaVO_4_:1Eu^3+^:0.05Cr^3+^ and LaVO_4_:1Eu^3+^:0.1Cr^3+^ phosphor samples.

A graph between [*αhν*]^2^*versus hν* also called as Wood and Tauc plot for different phosphor samples are shown in Fig. 8. The intercepts of the tangents on the curves on *X*-axis directly give the band gap. The band gap values for different concentrations of Cr^3+^ ions are given in the figure. This clearly shows that the optical band gap decreases with the increase in the concentration of Cr^3+^ ion, which is due to metallic nature of phosphor samples.^43,44^

#### 3.4.3 Photoluminescence excitation and emission spectra

**Fig. 9:**
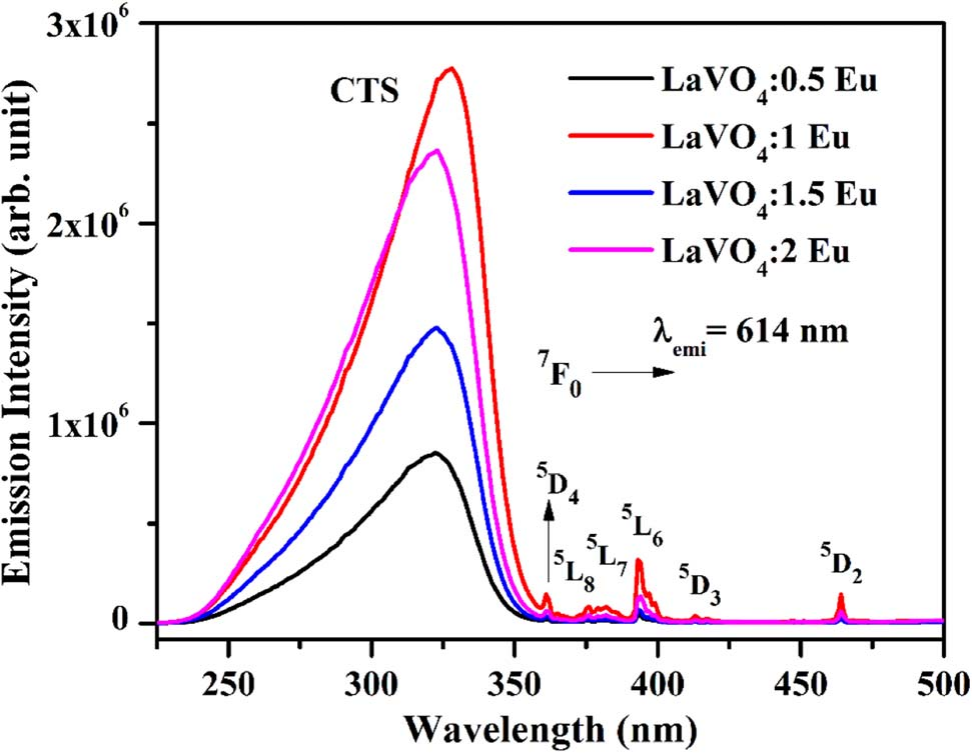
Photoluminescence excitation (PLE) spectra of LaVO_4_:xEu^3+^ (where *x* = 0.5, 1.0, 1.5, 2.0 mol%) phosphors monitored at *λ*_emi_ = 614 nm.

The photoluminescence excitation (PLE) spectra of LaVO_4_ doped with different concentrations of Eu^3+^ ions (*x* = 0.5, 1.0, 1.5, 2.0 mol%) have been monitored in the spectral reason of 200–575 nm with *λ*_em_ fixed at 614 nm. The PLE spectra thus obtained are shown in Fig. 9. A broad band is observed between 230–350 nm with its maximum at 316 nm. The maxima are slightly shifted towards higher wavelength side from 316 to 323 nm on increasing concentration of Eu^3+^ ion. The corresponding broad band is due to overlapping between two charge transfer bands (CTBs) *i.e.* (O^2−^ → V^5+^) and (O^2−^ → Eu^3+^). The spectra also contain many sharp weak peaks at 362, 376, 381, 393, 413 and 464 nm, which are due to ^7^F_0_ → ^5^D_4_, ^7^F_0_ → ^5^L_8_, ^7^F_0_ → ^5^L_7_, ^7^F_0_ → ^5^L_6_, ^7^F_0_ → ^5^D_3_ and ^7^F_0_ → ^5^D_2_ transitions of Eu^3+^ ion, respectively.^11–23,32,33^ The intensity of these peaks follows the trend *λ*_393_ > *λ*_464_ > *λ*_362_. The peak at 393 nm lies in the region of n-UV LED; therefore, it is very important. The intensity of charge transfer band (CTB) is much larger than the intensity of discrete bands due to 4f–4f transitions of Eu^3+^ ion. The intensity of excitation band is optimum for 1 mol% concentration of Eu^3+^ ion.

**Fig. 10:**
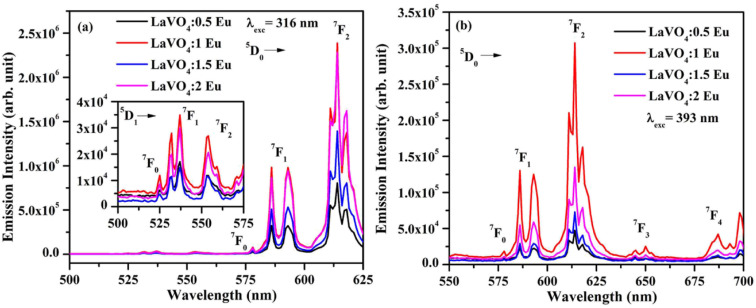
Photoluminescence (PL) emission spectra of LaVO_4_:xEu^3+^ phosphors on excitation with (a) 316 nm and (b) 393 nm (where *x* = 0.5, 1.0, 1.5, 2.0 mol%).

We have monitored the photoluminescence (PL) emission spectra of LaVO_4_:xEu^3+^ (where *x* = 0.5, 1.0, 1.5 and 2.0 mol% Eu^3+^) exciting at 316 and 393 nm wavelengths in the range 500–800 nm. In all the cases, we have observed intense bands in between 520-700 nm regions due to ^5^D_0_ → ^7^F_*J*_ transitions.^11–23,32,33^ The intensity of emission bands on excitation with CTB wavelength is nearly 10 times larger than on excitation with discrete bands of Eu^3+^ ion *i.e.* at 393 nm. The photoluminescence emission spectra on excitation with these wavelengths are shown in Fig. 10a and b.

As mentioned earlier, the bands observed between 570–700 nm are due to ^5^D_0_ → ^7^F_*J*_ transitions of Eu^3+^ ion in which the band at 614 nm due to ^5^D_0_ → ^7^F_2_ transition is very intense. The other observed bands are due to ^5^D_0_ → ^7^F_0_ (579 nm), ^5^D_0_ → ^7^F_1_ (582 and 591 nm), ^5^D_0_ → ^7^F_3_ (644 nm) and ^5^D_0_ → ^7^F_4_ (700 nm) transitions.^11–23,32,33,45,46^ The intensity of the bands follows the ^7^F_2_ > ^7^F_1_ > ^7^F_4_ > ^7^F_3_ > ^7^F_0_ trends. Though the excitation wavelengths (316 and 393 nm) populate high lying levels of Eu^3+^ ion; however, due to closeness of the energy levels, the excited Eu^3+^ ions relax rapidly to low lying ^5^D_1_ and ^5^D_0_ excited states. It is due to this reason that the observed emission bands are arising from these two lower lying levels. The weak emission bands in the green region are due to ^5^D_1_ → ^7^F_0_ (525 nm), ^5^D_1_ → ^7^F_1_ (537 and 531 nm) and ^5^D_1_ → ^7^F_2_ (565 nm) transitions, which are reported only in few host materials.^46,47^ The emission bands from ^5^D_1_ level is observed only on excitation with 316 nm wavelength.

The ^5^D_0_ → ^7^F_2_ transition occurs due to change in electric dipole moment. Its intensity depends on the external conditions. However, the ^5^D_0_ → ^7^F_1_ transition is magnetic dipole allowed transition and it is independent of the local environment round the Eu^3+^ ions. The ^5^D_0_ → ^7^F_3_ has an electric and magnetic dipole mixed character. On the other hand, ^5^D_0_ → ^7^F_4_ is forced electric dipole transition. Thus, the electric dipole allowed transitions have larger intensity compared to the magnetic dipole allowed transitions.^11–23,32,33^ Actually, the doping of Eu^3+^ ion in the LaVO_4_ host replaces La^3+^ ion and forms EuO_9_ instead of LaO_9_ in the case of monoclinic structure of LaVO_4_ host. The EuO_9_ in LaVO_4_ host forms a highly asymmetric structure, which is the basic reason for large PL intensity.

#### 3.4.4 Effect of Cr^3+^ doping on PL intensity

**Fig. 11:**
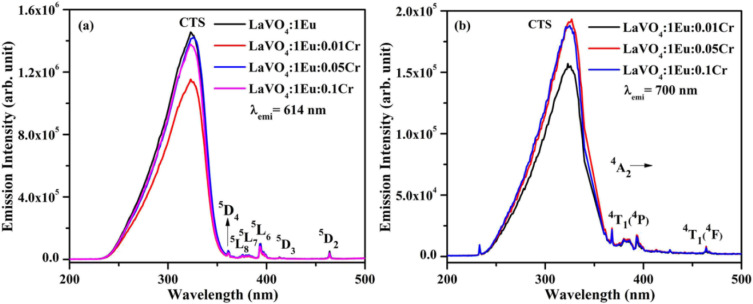
Photoluminescence excitation (PLE) spectra of LaVO_4_:1Eu^3+^:yCr^3+^ (where *y* = 0, 0.01, 0.05, 0.1 mol%) phosphors monitored at *λ*_emi_ = (a) 614 nm and (b) 700 nm.

The PLE spectra of LaVO_4_:1Eu^3+^, LaVO_4_:1Eu^3+^:0.01Cr^3+^, LaVO_4_:1Eu^3+^:0.05Cr^3+^ and LaVO_4_:1Eu^3+^:0.1Cr^3+^ phosphor samples have been monitored by fixing *λ*_emi_ = 614 and 700 nm. The Cr^3+^ ion gives an intense emission at 700 nm due to forbidden ^2^E → ^4^A_2_ transition. There is also a weak ^5^D_0_ → ^7^F_4_ transition due to Eu^3+^ ion at the same wavelength. We have monitored the PLE spectra taking *λ*_emi_ = 700 nm just to see, is there any other excitation band due to Cr^3+^ ion in this region. The PLE spectra of the samples for *λ*_emi_ = 614 and 700 nm are shown in Fig. 11a and b. The PLE spectra appear almost identical in the two cases though there is a decrease in the PLE intensity for *λ*_emi_ = 700 nm. An additional peak also appears near 386 nm, which have been taken due to Cr^3+^ ion as there is a band due to Cr^3+^ ion at this wavelength. The position of peaks remains unchanged; however, the intensity of the peaks varies with the concentration of Cr^3+^ ion.

**Fig. 12:**
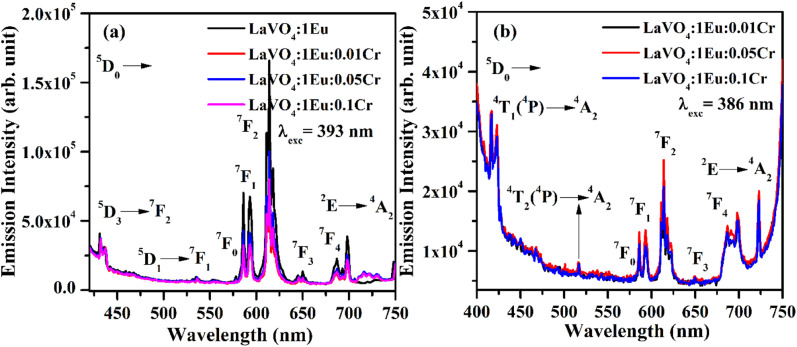
(a) PL emission spectra of LaVO_4_:1Eu^3+^, LaVO_4_:1Eu^3+^:0.01Cr^3+^, LaVO_4_:1Eu^3+^:0.05Cr^3+^ and LaVO_4_:1Eu^3+^:0.1Cr^3+^ phosphor samples on excitation with 393 nm and (b) PL emission spectra of LaVO_4_:1Eu^3+^:0.01Cr^3+^, LaVO_4_:1Eu^3+^:0.05Cr^3+^ and LaVO_4_:1Eu^3+^:0.1Cr^3+^ phosphor samples on excitation with 386 nm.

The PL spectra of LaVO_4_:1Eu^3+^, LaVO_4_:1Eu^3+^:0.01Cr^3+^, LaVO_4_:1Eu^3+^:0.05Cr^3+^ and LaVO_4_:1Eu^3+^:0.1Cr^3+^ phosphor samples were monitored on excitation with 393 and 386 nm and they are shown in Fig. 12a and b. The PL spectra of these phosphor samples in the range of 420–750 nm show similar emission bands on excitation with 393 nm (see Fig. 10b). However, some additional emission bands are observed at 431 and 536 nm, which are assigned to arise due to ^5^D_3_ → ^7^F_2_ and ^5^D_1_ → ^7^F_1_ transitions of Eu^3+^ ion, respectively. The emission intensity of Cr^3+^ doped LaVO_4_:1Eu^3+^ phosphor is slightly reduced on excitation with these wavelengths. It is also clear from Fig. 12a that the pure LaVO_4_:1Eu^3+^ phosphor does not contain any emission band due to Cr^3+^ ion. When Cr^3+^ ion is doped in the LaVO_4_:1Eu^3+^ phosphor; an additional broad emission band is observed in the range of 700–735 nm, which occurs due to ^2^E → ^4^A_2_ transition of Cr^3+^ ion.^34,38^ This additional band alongwith the other emission bands are clearly observed by exciting the phosphor samples at 386 nm in the range of 400–750 nm. In this case, the PL spectra contain additional broad emission bands at 417, 518 and (700 and 725 nm) clearly due to Cr^3+^ ion with weak PL intensity (see Fig. 12b) and they are assigned to arise due to ^4^T_1_(^4^P) → ^4^A_2_, ^4^T_2_(^4^P) → ^4^A_2_ and ^2^E → ^4^A_2_ transitions of Cr^3+^ ion, respectively.^34,38^

The LaVO_4_:1Eu^3+^ phosphor gives poor PL intensity due to the reason that 386 nm wavelength also excites the Eu^3+^ ion weakly. However, the PL intensity of 614 nm band is relatively larger than that of 700 nm band. The increasing concentration of Cr^3+^ ion reduces the PL intensity of Eu^3+^ as well as Cr^3+^ emission bands. This is due to energy transfer from Eu^3+^ to Cr^3+^ ions. Actually, the energy level ^4^T_2_(^4^F) of Cr^3+^ ion lies in the red region at 15 773 cm^−1^ while that of ^4^T_1_(^4^F) lies in the green region at 17 211 cm^−1^. On the other hand, the ^5^D_0_ level of Eu^3+^ ion is found to lie at 17 267 cm^−1^. The ^4^T_1_(^4^F) level of Cr^3+^ ion lies close but slightly below to the ^5^D_0_ level of Eu^3+^ ion. On excitation of Eu^3+^ ion, a small amount of energy is transferred to Cr^3+^ ion, which decreases the PL intensity of Eu^3+^ ion.^47,48^ This clearly shows that the Cr^3+^ ion works as intensity quencher even for smaller concentration of Cr^3+^ ion. This can be well reflected in the lifetime measurements as the lifetime of ^5^D_0_ level of ^5^D_0_ → ^7^F_2_ transition at 614 nm is expected to decrease with the concentration of Cr^3+^ ion.

#### 3.4.5 Lifetime measurements

**Fig. 13:**
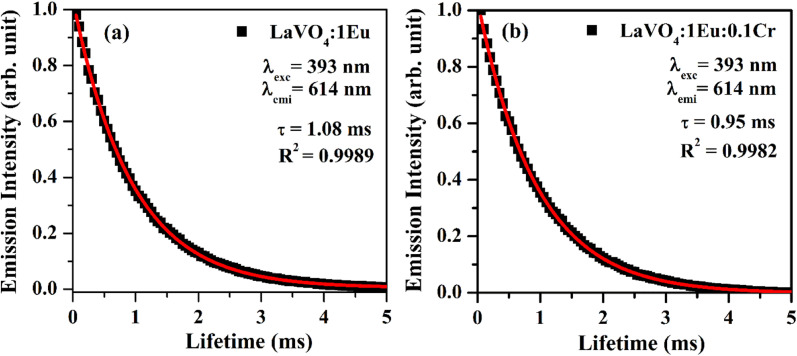
Fluorescence decay curves of (a) LaVO_4_:1Eu^3+^ and (b) LaVO_4_:1Eu^3+^:0.1Cr^3+^ phosphor samples for ^5^D_0_ → ^7^F_2_ transition on excitation with 393 nm.

The lifetime of ^5^D_0_ level of Eu^3+^ ion corresponding to ^5^D_0_ → ^7^F_2_ transition at 614 nm has been measured in absence and presence of Cr^3+^ ion. The sample was excited by 393 nm radiation and the fluorescence decay of LaVO_4_:1Eu^3+^ and LaVO_4_:1Eu^3+^:0.1Cr^3+^ phosphor samples are shown in Fig. 13a and b. We have tried to fit the decay curves using single exponential relation:^32,33^v
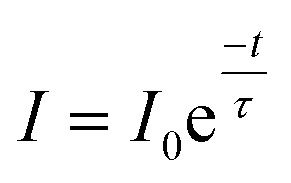
where, *I* is the emission intensity at time *t* and *I*_0_ is the initial intensity, and *τ* is the lifetime of ^5^D_0_ level. It was found that the single exponential relation does not reproduce the decay curves correctly and the error limit was found large. We therefore fitted the decay curves using double exponential relation:^34^vi
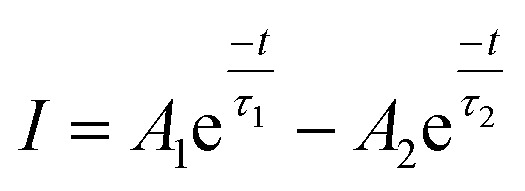


As is clear from the Fig. 13, the double exponential fitting reproduced the decay curves in a better way. The average lifetime was calculated using the following relation:^34^vii
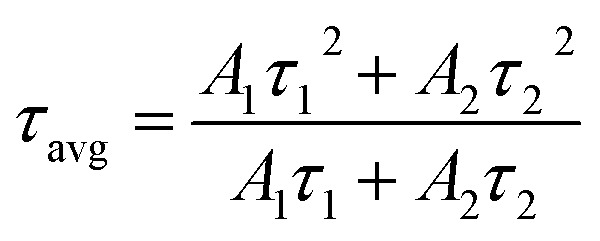


We have measured the lifetime of ^5^D_0_ level of Eu^3+^ in the case of LaVO_4_:1Eu^3+^ and LaVO_4_:1Eu^3+^:0.1Cr^3+^ phosphor samples. It was found that the lifetime of the ^5^D_0_ level decreases with the addition of Cr^3+^ ion. This also supports the energy transfer from Eu^3+^ to Cr^3+^ ions,^48^ which quenches the PL intensity of the phosphor.

#### 3.4.6 Heating effect under 980 nm excitation

We have also studied the heat generated in the LaVO_4_:1Eu^3+^, LaVO_4_:1Eu^3+^:0.01Cr^3+^, LaVO_4_:1Eu^3+^:0.05Cr^3+^ and LaVO_4_:1Eu^3+^:0.1Cr^3+^ phosphor samples on excitation with different pump power densities of 980 nm diode laser for 5 minutes. The idea is to see the heat generated in the sample because of low phonon frequency of LaVO_4_ host. The samples were prepared in the form of discs of 2 mm diameter and of ∼0.5 mm thickness. The disc was kept on an aluminum foil. The laser beam was focused on the disc in such a way that its spot size is of ∼1 mm radius on the disc. The heat generated in the samples was measured at a distance of 2 mm from the edge of the disc. The laser power density was varied from 0–64 W cm^−2^. It is well known that the heat generated in the samples will be maximum at the point where the laser beam is falling on the phosphor sample.^34^ However, it is difficult to measure it at that point due to sparking. If the heat generated is sufficiently large it can be used for localized thermal massage (physiotherapy) or controlled heating of the substances including water.

**Fig. 14:**
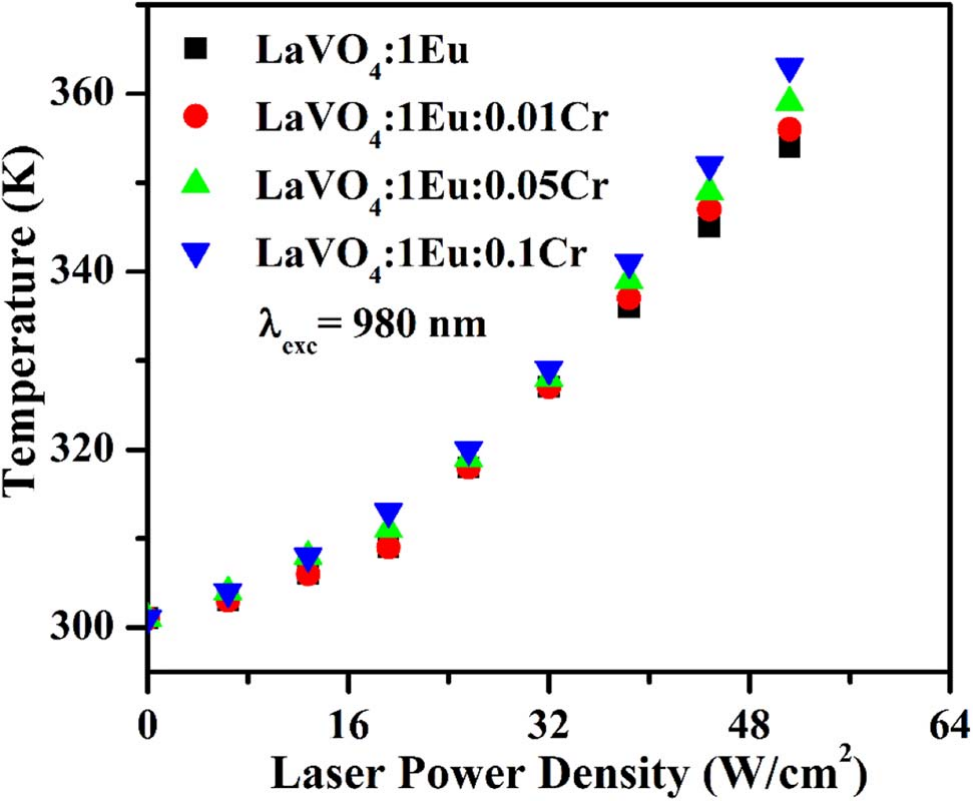
A plot of generated temperature *versus* laser power density for the LaVO_4_:1Eu^3+^, LaVO_4_:1Eu^3+^:0.01Cr^3+^, LaVO_4_:1Eu^3+^:0.05Cr^3+^ and LaVO_4_:1Eu^3+^:0.1Cr^3+^ phosphor samples on excitation with 980 nm.

The heat generated in the phosphor samples at different power densities of 980 nm is shown in Fig. 14. In the case of pure LaVO_4_ phosphor, the temperature generated is 336 K at 64 W cm^−2^ power density.^34^ This value is increased to 354 K at 51.2 W cm^−2^ in the case of LaVO_4_:1Eu^3+^ phosphor. Above 51.2 W cm^−2^, the phosphor sample starts burning. The heat generated is increased further *via* Cr^3+^ doping and it increases with the increase of concentration of Cr^3+^ ion. We have measured the heat generated in the LaVO_4_:1Eu^3+^, LaVO_4_:1Eu^3+^:0.01Cr^3+^, LaVO_4_:1Eu^3+^:0.05Cr^3+^ and LaVO_4_:1Eu^3+^:0.1Cr^3+^ phosphor samples on excitation at different power densities. The heat generated in the samples also depends on the NIR radiation absorbed by the samples. Since the Eu^3+^ and Cr^3+^ ions absorb the NIR radiation it may also be contributing to the heat generation. The brown color of these phosphor samples is an additional feature for generating large heat. A higher heat is generated in the colored phosphor samples, which is due to excitation of thermal phonons.

## 4. Conclusions

The Eu^3+^, Cr^3+^ doped and co-doped LaVO_4_ phosphors were synthesized by high temperature solid-state reaction method. The powder XRD patterns of phosphors are of crystalline nature. The particles of phosphor samples are spherical with their size in the sub-micron to micron range. The La, Eu, Cr, V and O elements are present in the Eu^3+^, Cr^3+^ co-doped LaVO_4_ phosphor. The FTIR spectra show the absorption bands due to La–O and V–O groups. The optical band gap of phosphor decreases on increasing concentration of Cr^3+^ ion. The Eu^3+^ doped LaVO_4_ phosphor was excited at 393 and 316 nm wavelengths, which gives intense red color at 614 nm due to ^5^D_0_ → ^7^F_2_ transition of Eu^3+^ ion. When the Cr^3+^ ion is doped in the Eu^3+^:LaVO_4_ phosphor; the emission intensity of phosphor is found to decrease due to energy transfer from Eu^3+^ to Cr^3+^ ions. The lifetime of ^5^D_0_ level of the Eu^3+^ ion also decreases in the Eu^3+^, Cr^3+^ co-doped LaVO_4_ phosphor due to energy transfer. The Eu^3+^, Cr^3+^ doped/co-doped LaVO_4_ phosphors also produces heat under 980 nm excitations. Therefore, the Eu^3+^, Cr^3+^ co-doped LaVO_4_ phosphor may be used for LEDs and heat generating devices.

## Conflicts of interest

There are no conflicts of interest to declare.

## Supplementary Material
